# Medical and Surgical History of the British Army Which Served in Turkey and the Crimea, (Blue Book)

**Published:** 1859-09

**Authors:** 


NORTH AMERICAN

### MEDICO-CHIRURGICAL REVIEW.

SEPTEMBER, 1859.

### Analytical and Critical Reviews.

ART. I.—*Medical and Surgical History of the British Army which served in Turkey and the Crimea, during the War against Russia, in the Years* 1854–55–56. 2 vols., pp. 560 and 480, folio. London, 1858.

LIKE most of the "Blue Books"* which are printed by authority of Parliament, and which are often the result of parliamentary inquiries, through committees appointed for the purpose, the volumes whose title heads this article convey a great deal of information on the matters of which they specially treat. Every one has heard of the war between Russia on the one side, and France and England, and, after a time, Sardinia, on the other. The Crimea and Sebastopol became familiar as household words, and almost equally wide-spread was a knowledge of the shortcomings of the British government at home, and of many of its official agents at the seat of war, respecting a mismanaged commissariat and the consequent sickness and mortality among the British troops, especially in the winter of 1854–55. There were certainly blunders on a large scale committed by these parties during the first year of the war; but, at the same time, it must be admitted that, great as were the sufferings of the British army from disease before Sebastopol, they were, as we learn from subsequent and semi-official accounts, fully equaled, if not exceeded, by those of their French allies, a fact which we shall probably take occasion to demonstrate before long.

---

* Some of our readers may not be aware that the designation of these booksi derived from the color of their paper cover. All of the volumes printed under au thority of Parliament wear the same livery.

The first of the two volumes before us contains the "Military History of Individual Corps" of the army, and an Appendix in which are presented reports on the climate, medical topography, and diseases of the provinces of Turkey, which were either the actual seat of war, or would be traversed by the troops in their expected march to the Danube. To these documents are added returns, showing the stores and other materials of the commissariat, including "Medical Comforts shipped for the Hospitals in the East," and a list of Hospital and of Medical and Surgical Stores sent to the same quarter; also, the "Names and Period of Service of all Officers who served in the Hospital Staff in the East."

The second volume is in two Parts. I. The "History of Disease," the several sections of which make us acquainted, first, with the topography and climate of Bulgaria, a Turkish province, and next, of the Crimea. To these sections succeed others, giving a general sketch of the causes of disease in the army and in the different sections of the service, then separate accounts of cholera, diseases of the bowels, fever, scurvy, frost-bite and gangrene, diseases of the organs of respiration, mortality, invaliding of the army on account of disease, and discharge of men from the service in consequence of disease incurred during the war. Part II., being a "History of Wounds and Injuries," contains, after an introduction, a section headed "General Observations," a second on specific wounds and injuries, a third on amputations and resections, and a fourth, entitled "Concluding Observations." An Appendix furnishes tables of meteorological observations, and returns of the sick and wounded who arrived at Scutari, and at Abydos, Smyrna, and Renkoi, or who died on board the transport ships between these several places; the whole being terminated by general hospital returns.

Having placed our readers in possession of the table of contents of the two volumes of "Blue Books," we shall next point out the subjects which will chiefly engage our attention in the present article. Our business will be with the "Medical History of the British Army," in the three years already mentioned; leaving the surgical part to be treated of on another occasion. We shall not attempt to repeat the details, or even to give an analysis of the military medical history of individual corps, which make up the larger part of the first volume; we may revert to it as occasion requires, when speaking of the diseases separately, as described in the second volume. There will still be a large field left to us for profitable gathering of materials which have an abiding interest to the medical practitioner, exhibiting, as they do, features in common and often identical with those which he is called upon, almost daily, to scan.

We begin with the medical topography of the provinces already referred to, as we find it in both volumes. The ground here is entirely new, and as the descriptions are given in a work inaccessible to the great body of our profession, we may properly anticipate a favorable reception of our extracts and summaries. Servia, though governed by its own prince, is still under the sovereignty of the Turkish Sultan, to whom it pays an annual tribute of $300,000. Its northern boundary is the Saave and the Danube; it touches Bulgaria on the east, and is separated from Bosnia on the west by the Dwina, while on the south it is bounded by a portion of the mountain range which extends from the Gulf of Venice to the Black Sea, and the eastern part of which is the Balkan proper.

"Servia is a mountainous country, well wooded, productive, with a rich soil, and a climate severe in winter, but warm in summer, ripening the grape, to be capable of giving rich, generous, and wholesome wines. The grain principally grown is maize. Herds of cattle are not numerous; hogs, fed almost entirely on the acorns of its vast forests, are largely exported from this country. Agricultural produce is conveyed on rudely-made bullock-drawn carts. The population amounts to one million."

The province of Bulgaria stretches from west to east, in a length of three hundred and fifty miles, between the Danube on the north, and the Balkan (the Mount Hæmus of antiquity) on the south; it touches Servia on the west, and its eastern side is washed by the Black Sea. This province, as described by Dr. Dumbreck, Deputy Inspector-General of Hospitals, may be divided into three portions: "First. The western Danubian portion, consisting of the often swampy banks of that river, rising gently and at a little elevation; vast, treeless, bare, scarcely cultivated pastoral plains, scantily supplied with water; the capital of this is Widdin. Second. The mountainous district, stretching from west to east, from near Nissa, in Servia, across the country to Tirnova and Shumla, which, with Sophia, are its great towns. Third. The districts of the Dobrinja, a peculiar tract at the northeastern end of Bulgaria, consisting principally of steppe-like country, characterized by low undulations, and the rank nature of its grassy vegetation, which attains an extraordinary height. This portion of the country may be regarded as most insalubrious. The principal sea-port, Varna, is the great town of this district."

Dr. Hanbury, author of the "History of Disease," in volume second, gives also a sketch of the topography and climate of Bulgaria, in this volume. Of the numerous mountain ranges extending northward from the Great Balkan, that which is of most immediate interest to us, on the present occasion, is one which springs from a point almost due north of Adrianople, stretches in a northwestern direction toward Shumla, and thence extends, though in a less continuous and regular manner, as a chain of hills of considerably diminished altitude, to the eastward as far as Varna. To the district bounded by this mountain range on the west and north, the Black Sea on the east, and the Balkan on the south, were the movements of the British army limited. The peninsula of Dobrudscha, of which so much was said in the public papers during the first year of the war, is described by Dr. Hanbury to be the most insalubrious part of the country, in its being flat, swampy, and liable to inundations. How are we to reconcile this with the description of the same district in Murray's Hand-Book of Turkey, in which the Dobrudscha is represented to be "a wilderness; its soil porous limestone, which retains no water, and furnishes no springs on the surface? Drinking water is obtained through a few deep wells. This desert extends south of the wall of Trajan, nearly as far as Basirijik and Varna." Our puzzle is increased if we are to suppose that this is the same district which Dr. Dumbreck calls the Dobrinja, noticed above. Its topography corresponds closely with the Dobrudscha.

Varna was the headquarters for a time of the British and French *corps d'armée* which were intended to co-operate with the Turks in their campaign on the Danube, or, if necessity required, in case of disaster, in their retreat to and occupation of Shumla and the passes of the Balkan. Varna, with a population of upwards of 15,000, is situated on the northern side of a small bay of the Euxine or Black Sea, in 43° 12′ north latitude, and 27° 55′ east longitude. The south or opposite side is formed of a lofty promontory three hundred feet high, and rising almost perpendicularly from the sea. It is called Point Galata Bournu, and is continuous with a chain of lofty hills thrown off from the eastern extremity of the Balkan Mountains, north of Cape Emineh, and which skirt the sea-coast for a distance of twenty miles. The streets of Varna run in tortuous confusion from east to west. The habits of the people are extremely filthy; and although drainage from some of the public buildings exists, it is imperfect and ineffectual. Odors and impurities of every kind defile the streets. The condition of the town was, however, much improved for a time by the French, for as soon as their *état-major* was established in Varna, large parties of soldiers and *gens d'arme* were employed in removing the filth, and many hundreds of cart-loads of what would have proved excellent manure were carried away from the principal thoroughfares. The harbor is about three and a half miles wide at its mouth; its depth inland is nearly three miles. At the head of the harbor the River Devna empties itself.

With the course of this river and the Pravadi, with which it joins at a distance of about four miles from its boggy sources, the most interesting features in the topography of that part of Bulgaria, as far as the army was concerned, is intimately connected. The river forms in its course, through a valley or plain, two lakes, the Upper and the Lower Devna; the first commencing at a point twelve miles from Varna, and the second, and the larger of the two, extends to within half a mile of the town; its extreme length is five miles, and its average breadth is less than a mile. Many parts of the valley are represented to be very marshy, especially on the borders of the lower lake, to the east of Alladyn. On the opposite side of the harbor, between the lake and water of the bay, there is an extent of marshy ground. Higher up, near the junction of the Devna with the Pravadi, there is an extensive swamp.

The climate of Bulgaria is determined more by the physical character of the province than by its latitude, which is between 42° 50′ and 44° 20′ north. Its position between the range of the Balkan Mountains on the south, and the broad expanse of the Danube on the north, and beyond it, of the still more distant Carpathian Mountains, disposes the wind to pass as a current from east to west, or the reverse. In winter, with northerly winds, the thermometer falls at times to 30° below zero, while in summer the prevailing winds, which are from the south, are often attended with a very elevated range of temperature,—in the tents, 100° F. Great and frequent transitions occur both in winter and summer. The spring is extremely short, for almost as winter ends, the summer heats are ushered in. The summer may be said to begin in April, and to last to the middle or end of September. The autumn, like the spring, is very short, and the severity of winter is usually felt in the middle of November. Autumn and winter are the seasons of rain. The sudden reduction of temperature during the night, in summer and autumn, to the extent, toward the evening, of 15° or 19° F., causes a heavy dew to form; and in the early mornings, the valleys are for many hours filled by dense clouds of fog, which roll into them along the hill-sides during the night. Dr. Linton portrays still more contrasted features of the climate of Bulgaria than those just presented. It is in vain, he says, to seek for a definition of this climate, and its nature can only be estimated by its effects; for, although a consumptive patient might, in some years, do as well here as in Algeria or Egypt, yet in others he would suffer as severely as on the east coast of Scotland. The finest morning in April, with almond-trees in full blossom, and nightingales scarcely hushed from their night-long melody, may soon be overcast, and the evening close in with a snow-storm; and the bright, clear frost of January is frequently thawed by a heavy sirocco wind, which raises the thermometer 20° in an hour.

The principal diseases of Bulgaria are, in the summer and autumn, low remittent and intermittent fevers, which are most prevalent in the district of the Dobrudscha and the marshy banks of the inland streams. On the approach of winter the fever becomes more of an adynamic type, and as the season advances, catarrhal and rheumatic affections, and typhoid pneumonia, are common; the period of spring and the first few months of summer are, in general, remarkable for the great immunity from disease which then prevails. The difference of temperature between day and night in July and August was the most extraordinary feature of the climate; and there can be no doubt, in Dr. Hanbury's opinion, that, attended as it was by such a heavy fall of dew, it was this which proved particularly injurious to the troops. In the actual state of our knowledge, we can hardly go farther than this, in the way of etiological explanation, without resorting to the mystic agency of malaria. Descriptions are given of the positions, in a sanitary point of view, occupied by the different divisions of the British army while stationed in Bulgaria. These were, in the vicinity of Varna, and on the shores of the larger Devna Lake; at Alladyn, on a low, wooded ridge, on the banks of the stream connecting the two lakes; at Devna, along the river, and on the shores of the lakes; and at Monastir, an elevated plateau 2300 feet above the sea level, to the southwest of Devna. We shall not follow our text in enumerating the different spots in this region which were occupied by different corps of the army before their embarkation in the latter part of August, 1854, for the Crimea. It appears that one of the objects of primary consideration in the selection of ground for an encampment was the readiness for procuring a supply of water. This would be more particularly the case with the cavalry regiments. The supply was better and more abundant on the heights to the north and south of the Devna lakes and river than in the neighborhood of Varna or of the village of Devna.

Dr. Dumbreck, in his itinerary and topography of the localities visited by him, speaks of Belgrade, the capital of Servia. In all directions from this city, except inland, "we see a fever-producing country; the desolate-looking Banat of Temesvar stretching far on one side, and the low, undrained tract, lying between the Saave and Semlin, are both unhealthy. The Saave often escapes from its banks, and then we have part of the lower town under water. Then follow the fevers of the summer months, which prevail especially in this quarter of the city. The soldiers in the elevated citadel would be comparatively safe, but the sentries furnished to this district are exposed to the influence of its malaria, and thus contract periodic fever." One addition to the many anomalies in the miasmatic hypothesis of the etiology of periodical fevers, is furnished in the case of the Valley Maidanpeck, traversed by the River Peck. "This valley is rich in iron ore, and is thickly inhabited. Maidanpeck lies on an oblong square-shaped spot, closely hemmed in on all sides by precipitous mountains; no marshes, and the stream running through the village lively, with sound banks. Spite of no apparent excitants of disease, there is no place in Servia so unhealthy as this. There is usually a resident medical man, but at the period of my visit he was absent in ill health." Such a spot in the United States would not be left to the uncertain care of one medical man. Three physicians at least would rush in to fill up so good an opening. "Periodic fevers," continues Dr. Dumbreck, "are found here during nearly the whole year. From the middle of July to the end of September a most severe form of this prevails, of the tertian and quartan type. General dropsy, ascites, and splenic enlargement are most common here. The unhealthiness of this place is proverbial all over Servia; they talk of its white population lasting only from three to four years, but I could not have any authentic information on the subject. About four hundred of its inhabitants are engaged in procuring the iron ore; they suffer more than wood-cutters or other laborers."

Of Widdin, a most important Bulgarian city, we are told that it lies on a dead level, and that the Danube washes its northern walls. The elements of disease exist everywhere, and in excess beyond Dr. Dumbreck's former knowledge of Turkish towns.

"To persons inhabiting the cities of western Europe, even those the least famed for their cleanliness, the incredible filth of a Turkish town cannot be imagined; everything bears the marks of squalor and decay. A well-known author, Ranke, speaking of Widdin, says: 'Its fetid bazaar, and its streets strewn with putrid carcasses, round which the vultures swarm, tell plainly that the majority of its 20,000 inhabitants are Mussulmans.' And speaking of another town in Bulgaria, he says: 'But the stench of the air gives sufficient evidence of the presence of Mussulman habitations.'

"Fever, and the numerous ills depending on the want of sanitary and police measures, must always hover over these unclean abodes, and it is not to be wondered at if the sanitary state of Widdin is, while I write, of the most unsatisfactory kind."

The condition of the military (Turkish) hospitals, at Widdin, was very bad. The large surface of the interior court of the principal one was covered with stagnant water.

"The wards are large, containing [each] about sixty sick; they are boarded and matted. The men lie in double rows round three sides of the room. There are no bedsteads, but the bedding is good; the mattress of straw, thick and comfortable; above this a thin one of cotton. The sheets clean, the diets carefully prepared, but the atmosphere is electric with the elements of disease, from the want of ventilation, and from the stuffy fumes of the detestable 'mangal.'"

Other hospitals in the place were in a much worse condition. The air in all of them was laden with the seeds of disease. "Several of the medical men employed—fifty-six—had been attacked by the typhoid disease, but in their instance it was mild and manageable, for the very obvious reason that they were not treated in the poisonous atmosphere of the wards where they contracted the ill."

In relation to Turkish military hygiene, the following scale of hospital diet is not without interest:—

"1st. Rice, 13½ drachms, boiled in water.

"2d. Rice, 13½ drachms, and bread, 30 drachms.

"3d. Rice, 13½ drachms; bread, 50 drachms; meat, 30 drachms.

"4th. Rice, 13½ drachms; bread, 50 drachms; meat, 50 drachms.

"5th. Three days prior to the discharge, the barrack ration.

"The medical officer has it in his power to add to the fourth rate of diet any extra he thinks necessary—fish, wines, eggs, etc."

The treatment of disease by the medical men at Widdin, is represented to be rational and scientific; they deplete cautiously; they use calomel more freely than continental physicians do, or at least did twenty years ago. Vaccination is practiced in the army, and among the Turks generally. Dr. Dumbreck was told that it is carefully looked after in the army. He doubts much its being carried out extensively in civil Turkish communities.

Lom Palanka, at a distance of ten hours from Widdin, rises from the River Danube, over some irregular sand hills; it is said to be salubrious, for although periodic fevers are not unknown, they are neither frequent nor severe. The cause of these diseases "is referred to the too free use of fruit, but there is a much more evident reason in the appearance of a small alluvial plain to the eastward of the town; this is traversed by the River Lom, and is subjected to occasional inundations, and, of course, malaria follows." A large proportion of a cavalry regiment, about two thousand strong, stationed here, were suffering (March) from pulmonary diseases —pneumonia and bronchitis.

The Bulgarian peasant cannot promise himself either health or longevity from the dwelling which he occupies. It is constructed as follows: "A deep trench, like the foundation of a house, is dug; wattles form a pointed roof over this; earth is then thrown over the wattles, a hole is made for a chimney, and the house is completed: these dwellings are, therefore, mere burrows."

Tirnova, once the capital of the ancient Bulgarian kings, is a mountain city, most picturesquely situated on the bank of the River Yantra. It is a place of great trade and bustle, with a population of 10,000; but its streets, even in dry weather, were filthy in the greatest degree. Notwithstanding this drawback, it may be stated, for the encouragement of those who see a due affinity between health and dirt, the town and neighborhood are very healthy. This town is deemed to be the best fitted for hospital establishments on a large scale.

Shumla, which in a strategical point of view is so important, as guarding the chief passes of the Balkan on the north, has a population of 60,000. This mountain stronghold "is not free from fevers, amenable to treatment in spring, grave and tedious in autumn. Typhus is not infrequent in winter; filth, poor living, neglect of every sanitary precaution, are palpable enough reasons for the presence of this form of disease. The city is seething with every imaginable description of filth. The winds are high and keen here, and pulmonary affections are those from which the large force now in Shumla suffers."

Rumelia, the Turkish province separated from Bulgaria on the north by the Balkan Mountains, contains the two chief cities of European Turkey, viz., Constantinople and Adrianople. The height of the Balkan range is, in some places, upwards of 7600 feet, and the highest pass, "the Shibka," leading from Kazanlik to Gabrova, is 4350 feet above the level of the sea. The summits of the Balkan Mountains are generally covered with snow, and their slopes with oak, beech, and other wood of various kinds. The chief rivers of Rumelia are the Maritza, the Tundja, the Arda, the Vopoche, and the Stanimak. The Maritza, the ancient Hebrus, has its sources near Trajan's-Gate, in the northwest angle of the Balkan Mountains, flows through the centre of Thrace, and reaches Adrianople, whence, after receiving the Tundja and the Arda, it runs south and southwest to the Ægean Sea, and enters the Gulf of Enos, to form the port of Adrianople.

As relates to the climate of Rumelia, it may be described as nearly the counterpart of that of Bulgaria. Both provinces are marked, in this respect, by great extremes and sudden vicissitudes of temperature. The average quantity of rain which falls at Constantinople in the year, amounts to 361 parts in winter, 350 in autumn, 191 in spring, and 90 in summer. The north wind blows 167 days in the year, the south 63, and the northeast 47. The medium temperature throughout the year was nearly 60° F. (59·65.) The months which are marked by the two extremes are December, the mean of which is 47·25° F., and July, which is 75·79°. The barometer ranges within the narrow limits of 29·60 and 29·87 inches. Dr. Linton, to whom we are indebted for a knowledge of the climate and topography of Rumelia, is of opinion that the country, with few exceptions, is healthy; and such exceptions are chiefly confined inland, in the vicinity of the River Maritza and its tributaries, in a few localities where, forming marshes, it either overflows its banks, as at Adrianople, at Philippolis, and at Mustafa Pasha, or where it is brought into use for the purpose of irrigating large extent of rice grounds, as at Philippolis, at Tatar

Bazarjick, and at Buckilu, or where its banks are low or descaling, as at Papauslu, and at Mustafa Pasha. The people exhibit, generally, a robust, strong, and healthy appearance, and seem to be capable of enduring a large amount of fatigue. There is a remarkably small number of practicing physicians.

As to the prevalent diseases in Rumelia, we are told by Dr. Linton that the intermittent fevers of the country generally begin in June, and are followed during the months of July and August by remittent and typhus fevers, gastro-enterites, and sometimes dysentery; and that only some of the graver fevers are occasionally fatal. In most regions in which periodical fevers are met with, the remittent precedes the intermittent form. In the country now under notice, intermittent is said sometimes to be the precursor of petechial typhus, and remittent fever does not seem to be of such frequent occurrence as the other forms, on which continued fever sometimes supervenes. At Adrianople, with a population of 90,000 to 100,000, the greatest number of deaths occur during the winter months, from inflammatory complaints—catarrh, pneumonia, pleurisy, pericarditis, rheumatism, etc., complicated with typhus fever. The mortality among the military in this city is usually three per cent. When the Russians were there, in 1829, their loss by deaths was three hundred daily, chiefly from the plague. At Philippolis, the greatest mortality occurs from fevers, during the summer months, a fact to be attributed to the very great extent of swampy ground and rice cultivation, etc. in its vicinity. Immunity from fever can always be secured by a removal to the higher grounds during the summer months, a course which is almost invariably pursued by the wealthier portion of the population. Typhus petechialis is often met with here, and proves fatal in many cases. The intermittent fevers of the country generally appear in the tertian or quotidian form, and the practice usually adopted is to exhibit quinine in large and repeated doses till cinchonism is produced; and for the better securing the patient against a relapse, this medicine is continued in smaller doses for several days after all fever has disappeared. Quinine failing to produce the desired effect, arsenic is had recourse to. Sulphate of quinine and cyanuret of iron, in the proportion of two parts of the former to one of the latter, is sometimes prescribed. It is pleasant to see the wide diffusion of therapeutics founded on identical scientific observation and experience in countries so distant from each other, so that the Mussulman inhabitant of an interior province of Turkey undergoes the same treatment for periodical fever as the Christian denizen of the Mississippi Valley. In the inflammatory affections, blood-letting is carried to a considerable extent, which, together with purgative and emetic medicines, the people are said to bear well; but the latter less so at Constantinople than in the interior, a difference everywhere and always observable between the inhabitants of a large city and those of the country. Recourse is often had to leeches, which are readily procured, and are cheap. Epidemics of scarlet fever and of measles, and occasionally of smallpox, occur during the spring and summer months, as in other countries. Vaccination is said to be regularly performed, at least in all towns and in their immediate neighborhood, where any medical practitioner resides, as at Adrianople, at Philippolis, at Kaloferi, at Kirklissia, and at Tchorlu. Erysipelas is of frequent occurrence in the spring. Phthisis pulmonalis rarely occurs among the Turks, but it is occasionally seen among their black slaves. Scrofula, in damp and moist situations, is not uncommon. Madness, suicide, hydrophobia, elephantiasis, and goitre, are seldom seen. Stone in the bladder is rare, four cases only having been known to occur in Philippolis during a period of ten years. This city contains 70,000 inhabitants, of a mixed race. The intoxicating liquor called raki, the product of distillation from the husks of the grape, is drunk at Adrianople and other towns to a great extent: it sometimes produces delirium tremens and impotence. This latter effect was said by Larrey to be caused by a spirit obtained from the dates, which was drunk by the French soldiers in Egypt. Contrary to what had been previously advanced by Dr. Linton, respecting the general healthiness of this part of Turkey, he tells us, that few strangers escape either fever in the summer, or inflammatory affections in the winter. Such painful processes of acclimation would be thought a great hardship for European emigrants to undergo in Middle or Western Virginia, or in East Tennessee, regions analogous in their geographical features and isothermal lines to Bulgaria and Rumelia. There has been no plague in the country since 1842.

What remained to complete the outline view of that part of Rumelia between Adrianople and Constantinople, and between both of these cities and Enos, on the Ægean Sea, is furnished by Mr. J. Mitchell, staff surgeon, first class. The country between Adrianople and Constantinople is undulating; the soil rich in no ordinary degree; and there are no marshes, with very few exceptions of limited range. The distance between the two cities is one hundred and forty-five miles. Constantinople has a population of 700,000 to 800,000, and is on a level with the Sea of Marmora. The mortality is one in forty-six, annually. When fever prevails in this city its complications are usually abdominal. About two and a half per cent. of the strength of the military are usually in hospital at Constantinople. "Consumption is not common among the Turks, Greeks, or Armenians, but the negro race are, as in other countries where they are expatriated, singularly liable to it. Hydrophobia does not often occur, though Constantinople swarms with dogs." Phthisis is more frequently met with among the military than among civilians; and young recruits, who come from remote parts of the Turkish empire, are most subject to it. Of the months, October is the most healthy, and August the most unhealthy. Mr. Mitchell believes the hospital accommodations at Constantinople to be abundant. In the event of pressure of the sick and wounded, the very ample and spacious corridors would afford additional space for beds. The mosques alone would afford accommodation for 100,000 men—an appropriation of these edifices which would not be looked upon with much complacency by the faithful Mussulman.

Under the head of "Camps and Positions," we gather information which harmonizes with the accounts of nearly all travelers in both European and Asiatic Turkey. The hydrological appliances described might well be imitated in Christian lands.

"The numerous high ridges and valleys which intersect the line from Constantinople to Adrianople offer very many favorable positions for camping, either temporarily or otherwise. There are fountains of good water at short distances along the roadway, besides those in every town and village. The water is conveyed to the fountains in all cases through earthen pipes. The Turks pay a sort of religious attention to these fountains, as an act of charity for travelers as well as an act of necessity for themselves. The fountains never fail in a good supply of water, except when the pipes get out of order, which is a thing of rare occurrence."

Enos, already mentioned as at the mouth of the Maritza River, is ninety-six miles south from Adrianople, and one hundred and fifty-four southwest from Constantinople. This town contains 10,000 inhabitants, who, in general, have a healthy appearance. It suffers lightly from intermittent fever in July and August, which is seldom followed by dropsies, visceral enlargements, or indeed by any subsequent deterioration of health.

On the Topography and Climate of the Crimea, our notes and extracts cannot be as full as would be justified by the latitude of description in the first part of the second volume of the History now before us. The peninsula of the Crimea is situated between 44° 20′ and 46° parallels of north latitude.

"The country is topographically divided into two portions; about three-fourths of the peninsula toward the north and west represent a vast elevated plain. This extensive plateau or steppe is, for the most part, dry and sandy, and the soil is either barren or barely fit for the purposes of pasturage, and a large district toward the northwest is impregnated with saline matters. Along the sea-coast lakes are frequently observed, while from the waters about the isthmus of Perekop large quantities of salt are annually raised in the interests of commerce. The remaining or mountainous portion of the Crimea extends along the shores of the Black Sea from Sebastopol on the west, to Kaffa, or Theodosia on the east, and is about ninety-five miles in length, and from ten to eighteen miles in breadth. The principal range of hills extends in a direction from west to east, and it varies in height from one thousand to five thousand feet, the point of culmination being in the Tchatar Dagh, south of Simpheropol. The division forming the steppe is considerably above the sea level, and the coasts of the peninsula are of difficult access almost throughout. The district of the Crimea which was the scene of military operations during the late war comprises that portion of it forming the southwestern extremity of the peninsula, formerly called the Heracleatic Chersonese; its limits are found between 33° 20′ and 33° 46′ east longitude, and 44° 20′ and 45° north latitude. It is bounded on the north by the harbor and city of Sebastopol, on the northeast by a range of hills which, carried round from the western coast north of the harbor of Sebastopol, become more elevated in the Inkermann and Mackenzie Heights; on the east by the mountain range which stretches from Balaklava along the southern coast, and on the northwest and southwest by the sea. The district thus defined divides itself into two portions, a western and an eastern division; the former represented in an irregular barren plateau, of varying elevation, while the latter comprises the valleys of Balaklava and Tchernaya, the village and harbor of Balaklava, the heights overhanging Balaklava to the east, and the elevated ground in the vicinity of Kamara, to the northeast of Balaklava, looking down upon the valley."

The face of the triangular plateau, south of Sebastopol, has a general inclination to the northwest; from the sea in this elevation the ground rises gradually over a succession of undulating hills, flat downs, and shallow valleys; but proceeding southward from Sebastopol the ascent is much more abrupt, and follows a series of winding ravines and elevated ridges to a height varying between five and six hundred feet,—the general level of the position upon which the main body of the English army was encamped. A line drawn from the inner harbor of Sebastopol, due south to the Monastery of St. George, divides the plateau into an eastern and a western portion; the former, the site of the English occupation, is the most elevated; the latter, the western, was covered by the French troops, and slopes down toward the sea between Cape Chersonese and the mouth of the harbor, where the coast is indented by several shallow bays, often with low shelving shores. A deep ravine, running down to the inner harbor, marked the course of the northern portion of the line, and divided the English from the French camp. The position of the former before Sebastopol overlooked the great harbor on the north and the valley of the Tchernaya on the east. The village of Balaklava consists mainly of two streets, running parallel to each other and the bay, in different terraces, connected by cross lanes. A military school and a Greek church were its most prominent architectural objects. The harbor of Balaklava is a narrow inlet of the sea, in the southwest coast of the Crimea, formed by a breach in the southern mountain range. At its entrance it is extremely narrow; its greatest length is half a mile; for two-thirds of its extent it is very deep, but toward its head it expands, becomes shallow, and receives the water of a small stream which drains the Valley of Balaklava. The Marine Heights are a group of hills to the east of the town, on which a portion of the army was posted.

The geological basis of the allied occupation consists, to a great extent, of the limestone formation; on the plateau before Sebastopol the tertiary form is that on which it is chiefly exhibited; but to the eastward of the Monastery of St. George, and of a line extending thence toward Kamara, old mountain limestone and conglomerate are observed; while the hills which extend across the Valley of the Tchernaya abound in soft calcareous rock and green sand. This valley is watered by a river of the same name, the margins of which "are low and alluvial, and the river frequently overflows its banks for some distance toward the termination of its course; and there is reason to believe, that during the summer and autumn of 1855, fevers of the periodic type and of an adynamic kind prevailed to some extent among the French and Sardinian troops, produced by the malarious exhalations of the locality." The configuration of the ground, the plateau before Sebastopol, was highly favorable to natural drainage, and the rain which fell on it, except in a few isolated localities, rapidly ran down the hill-sides, or made its way into the ravines through the substratum of porous rock, and was thence carried into the harbor.

We find recorded in this section of the history, instances of the effects of very limited localities on the health of the troops encamped on them. The 42d and 79th Regiments (Highlanders) occupied an intrenched camp on the heights, and to the right of Balaklava. The camp of the first was dry, beautifully laid out and kept, and proved extremely healthy. The 79th was posted on higher ground, and, at first sight, would seem to have had a better situation for their camp than their fellows of the 42d; but there were several marshy spots and springs in the vicinity. The surface of the ground was always damp, and no amount of draining sufficed to correct this property. The foundation of the huts, owing to the exposed position and the dread of severe storms, increased this evil, as they were excavated some two or three feet. A deep trench was cut round each hut, leading to main drains. Each hut was calculated to accommodate twenty-five men. Provision was made for ventilation; but neither this nor white-washing and fumigations prevented the occurrence of fever, which only ceased after the removal of the regiment to higher and drier ground. And again, in the wing of the 2d battalion Rifle Brigade, which occupied the high promontory overlooking the sea at the southern extremity of the Balaklava lines, there was little or no disease. At a later period, the 31st Regiment was put into the huts which had been occupied by the 79th, and it was attacked with cholera, as were also two batteries of artillery and a small-arm brigade, which were encamped outside the lines, just above the place were the slope terminated in the plain, and where the soil was of the same nature. The whole encampment was moved to a knoll out on the plain, with the effect of an immediate improvement on the health of the men. One cause of impurity of the air (the privies) was prevented by the judicious location of these latter, such as at ravine-sides, etc., and the strewing of quick-lime with occasional intermixture of peat-charcoal. The graves were strewed with the same substances. Precautions of this nature were not uncalled for, as fever of a typhoid form appeared in the regiments occupying the low slope of the hill overhanging the large burying-ground of the Division. Contributing to the same morbid effects were the exhalations from the slaughtering ground. Purifications of this and the old burial-grounds, and selecting more distant localities in their stead, produced a removal of the disease. Diarrhœa prevailed to an extraordinary extent, and cholera continued its attacks from the first night to the last day of the encampment of the 10th Hussars in the Valley of Varnoutka, the low grounds of which were covered with a rank vegetation, tracts of marshy ground here and there, and it was shut in by mountains densely wooded. Of Balaklava, it is said that it embraced so many sources of impurity, accumulated mud and putrescent matter of every description, that it was utterly impossible to analyze the special influence of each, certain though it were that their conjoint action proved for a time disastrous and extensive in its effects.

On the climate of the Crimea, Dr. Logan makes the following observations :—

"The heat, during the summer solstice and in the course of the autumn, on the plateau before Sebastopol, rarely if ever exceeded 85° or 87°, and the nights were invariably cool and refreshing during these seasons. The principal characteristics of the region were the occasional occurrence of a summer storm, and frequent vicissitudes of rain and lowered temperature, features of climate everywhere appertaining to mountainous formations, and of which character Southern Crimea pre-eminently partakes.

"The autumn equinox this year was marked by wet and chilly weather, yet of not unusual duration.

"No more genial or healthy climate could there well be than that of October and the greater part of November, 1855, a blandness equal to that of Italy, in striking contrast with a portion of November of the previous year, so marked in the history of the campaign.

"The autumn did not break up before the twenty-first of November, but snow soon appeared on the mountain summits; hereafter set in boisterous weather, wet and inclement, varying with gales of violence from southeast and southwest until toward the middle of December; hard frost, followed by clear, bright skies and dry, intense cold, felt by night, set in, the thermometer down to zero in the open air, and at eight and nine degrees in a double-boarded hut, or twenty-five degrees below freezing point.

"These latter atmospheric conditions—and singularly healthy they were found—continued until the earlier part of January, when heavy falls of snow took place; then followed a winter of frequent variations of snow, thaws, and frost, and a great prevalence throughout of harsh and violent winds from the northeast and southwest, until a well-advanced period of spring, and altogether a great exemption from excess or duration of humidity, a most favoring circumstance toward the preservation of the excellent health the army had acquired, and which appeared almost proof against the many abrupt and extreme changes to which the Crimea, from the mountainous character of the southern region and its peninsular form of prominence on the Black Sea, is subject to.

"Early in April, 1856, spring established itself in extremely fine weather; May also proved a very fine month, light westerly and northwesterly winds prevailing; occasionally, however, subject to the humid atmosphere of low-spreading clouds—the phenomenon familiarly denominated the Black Sea fog, from its density and low stratum; a clear sky often showing immediately over it. Toward the latter part of May the temperature began rapidly to increase, and attained a maximum of 80° in a double-boarded hut at Kamara; the nights, however, being ever cool, and equally so from this period until the departure of the army; a month later the climate was most congenial to the health of the troops, the thermometer showing no wide oscillations, or a medium of temperature in the camp huts greater than 75°; light winds from westerly points were the most prevalent at this season, and were attended with occasional showers of refreshing rain."

Under the head of *Mortality* we find noticed its course in the army in general, the monthly proportions to the total mortality during the war; its course in the several arms of the service; in the several divisions of the army, and in regiments of infantry which joined the army after it arrived in the Crimea; and finally, among the officers of the army during the war. Tabular statements and diagrams elucidate these several topics.

The relatively small proportion of deaths from wounds in battle to those which are caused by different diseases, affords matter for serious study, and ought, on account of its moral aspects, to be universally known, as the best preventive, or, at any rate, refrigerant remedy against a war fever, which sometimes rages epidemically. It appears from a table, showing the number of men who died in each month from all causes, from wounds and from the principal classes of disease, that the mortality from wounds alone and mechanical injuries, from April, 1854, to June, 1856, was but 1761 out of 18,058, from all causes—diseases and wounds. The deaths from cholera were 4512 ;* from diseases of the bowels, 5950 ; from fever, 3446 ; from diseases of the lungs, 614 ; all other causes, 1745. The troops landed at different times in the Crimea amounted to nearly 48,000 (47,747) men of all arms. The entire number of admissions into the hospitals and hospital ships during the war was 162,673.

During the period the army was being assembled on the coasts of Turkey, viz., in the months of April, May, and June, 1854, the number of deaths which occurred among the troops amounted only to forty-one; and it appears that the mortality was limited exclusively to the infantry portion of the force. In July, cholera appeared in the camp, fever became more prevalent, and disease proved fatal in the proportion of 1·34 per cent. of strength in the infantry; 1·25 per cent. in the ordnance, and 1·13 per cent. in the cavalry; and while cholera proved most destructive in the infantry and cavalry, fever was the more mortal in the ordnance than in the other two branches of the service.

Fluctuations in the degree of mortality of the different branches of the service are noted. The predominance of mortality in the ordnance department, in August and September, was owing to fever, the result, doubtless, of residence in the filthy neighborhood of Varna and the exposure to the sun in the performance of heavy fatigue duties. In October the higher proportion of mortality, or 2·76 per cent. of the strength, was in the cavalry, and was caused by fever and cholera. The following exhibits the nature of the causes of disease and death which press most heavily on a besieging army in an inclement season, when deprived of the means of keeping up bodily vigor by appropriate food and protection against atmospherical extremes and vicissitudes :—

"In November, the measure on which the peculiar hardships of the siege began to bear on the several branches of the service, was first clearly indicated in the relative mortality which occurred; during the month of

* The author of the Report whose classification we follow, does not, it will be seen, recognize cholera among bowel diseases, or as a disease of the alimentary canal.

August, the proportion of deaths was greater in the cavalry and ordnance than in the infantry; and in September, the mortality in the ordnance was in excess of that of the infantry; while, in October, the ratio of casualties in the cavalry predominated over that in the infantry; and the difference of result was determined, not alone by the prevalence which cholera obtained, but by the extent to which 'fatigue duties,' exposure to the sun, and the special influence of locality, proved detrimental, in increasing the mortality of affections of the bowels, and particularly of fever; but in this month the number of deaths in the infantry represented 3·41 per cent., and in the cavalry and ordnance 1·68 and 2·26 per cent., and while a large proportion of the loss sustained by the infantry was the result of cholera, which assailed the regiments and drafts which arrived as reinforcements to that portion of the army, it was in part, also, caused by the more fatal character which the fluxes had began to assume among the infantry troops, as a consequence of the increasing labors and difficulties of the siege."

The next month bore evidence of the disproportional degree in which the rigors of the climate, the privations and the hardships of the service, were pressing on the different arms of the service; for whereas the number of deaths from diarrhœa and dysentery represented only 1·49 per cent. in the cavalry, and 2·01 in the ordnance, it amounted in the infantry to 2·90 per cent. of strength.

Still stronger was the evidence furnished of the force of the exposures to which the infantry, more than either of the other arms of the service, was subjected, and of the resulting mortality.

"In January, (1855,) the number of deaths exceeded that of any other month during the war, not only in the army but in the several arms of the service; yet the extraordinary mortality which the troops now sustained was almost entirely independent of cholera, (for that pestilence, after inflicting for many months great losses on the army, had at length nearly quite disappeared from the camp,) and was all but exclusively the result of the conditions of the service, as expressed in exposure, night-watching, duty, diet, accommodation in tents, bedding, etc., and the measure in which these conditions affected injuriously the different portions of the army, is explained in the statement, that the loss incurred by the infantry during the month amounted to 10·46 per cent. of strength; in the ordnance to 5·24 per cent., and in the cavalry to 3·95 per cent.; and that the total deaths from the fluxes alone represented in the infantry 6·98 per cent.; in the ordnance 3·82 per cent., and in the cavalry 2·23 per cent. of strength."

During a period which elapsed from the beginning of the winter in November, 1854, to the end of February, 1855, the proportion of deaths in the ordnance was considerably in excess of that in the cavalry, and in the infantry it was nearly twice as great as that in the ordnance.

In the month of April the diminution in the mortality was not only conspicuous in the army generally, but was almost equally so in every portion of it. Its increase, again, in the two months following, was owing to cholera, which reached its highest degree of epidemic violence in June. A decline in July was followed by an augmentation of deaths in August, a result attributable to cholera. In September, October, and November, the sanitary condition of the army was improved; but in December the mortality again rose.

The regiments of infantry which joined the army after it arrived in the Crimea, suffered greatly from disease, and the consequent mortality was excessive. One regiment, the 46th, which landed on the 8th of November, 1854, lost 533 men from disease alone, or the unparalleled proportion of 105·7 per cent. of mean monthly strength, and 36·6 per cent. of the total strength. The diseases which proved so largely destructive in all these regiments were cholera and diseases of the bowels. Of the whole number of deaths returned in the 46th regiment, 156 were caused by cholera, and 249 by fluxes, while 74 deaths were the result of fever, and 11 were assigned to diseases of the pulmonary organs. Part of the excessive mortality in the newly-arrived regiments was due to the abrupt manner in which these corps were introduced to the labors, hardships, and exposure of the siege. To these regiments, and the drafts which arrived about the same time, the reporter "would especially point for an illustration of the appalling ravages, which the direct action of the choleraic poison on men predisposed, and the severe application of the proper causes of the fluxes, were capable of producing in the ranks, independent of association (at least for a time) with scorbutic taint of the blood."

In reference to the mortality from disease among the officers of the army during the war, it was remarked that they suffered in less proportion than the private soldiers,—a fact corresponding with observations made in other parts of the world—East and West Indies, etc. The reasons for this difference in the Crimea were obvious enough, and are thus summed up by Dr. Marlow, (28th regiment.) "The extraordinary immunity of the officers from disease and death is extremely remarkable, and the circumstances, in my opinion, form the key to the explanation which may be offered of the almost unprecedented mortality among the men. It has been shown that the mortality in the former class amounted in the Third Division to little more than four per cent., while in the latter the mortality exceeded seventeen per cent.; and this will not be wondered at when it is remembered that the officers could nearly always command better food, clothing, and, in some respects, shelter at a time when the men were on short rations, with a very scanty supply of fuel, (for which they were indebted almost solely to their own exertions;) the officer was enabled either to procure fresh mutton, or fowls, or preserved meats, or soups, obtained from the ships at Balaklava; when the officer returned from the trenches, cold and drenched to the skin, he was certain to be provided with hot tea or coffee, and a tolerable supper, a change of clothes, and a reasonably warm bed; while the private, in many instances, had neither tea, coffee, nor supper of any description, but lay down in his wet, vermin-infected clothes, and equally moist blankets, in the mud, of which the floor of his leaky tent was composed. The duties of the soldier were infinitely more harassing than those of the officer, for the former, after returning from the trenches, was sent through the snow or mud to Balaklava for supplies of various kinds." This description is more particularly applicable to the state of things in the winter of 1854–55. By the time that the next winter had arrived, the condition of the army was every way improved, by the substitution of huts for tents, and the use of better clothing and more varied and nutritious food.

It appears from the estimates made on the subject of mortality from diseases in the army at different periods of life, that the very young and the old soldiers were the greatest sufferers. We learn from a diagram illustrating the mortality of six regiments for quinquennial periods of life, that the proportion of deaths in the first year of the war was greater between the ages of 16 and 20 than between 21 and 25; that it was greater between the ages of 21 and 25 than between 26 and 30; and that having reached its lowest point between 26 and 30, it increased slightly between 31 and 35 years, and then more rapidly; the greatest mortality having occurred above the age of 40. These conclusions would probably be modified by the previous period of service in the army. What, for example, would be the comparative chances of survivorship in two men aged forty, one of whom had served fifteen or twenty years, and the other who had just enlisted?

Bearing on this point are the remarks of Dr. Battersby in the section on "Invaliding in the Army on Account of Disease." It appears that the total number of men invalided in England amounted only to 9544. After alluding to the diseases under which the invalids chiefly suffered, Dr. Battersby, in reference to the relaxation of the rules for the examination of recruits, remarks:—

"Two classes of men were thus enlisted, of very different stamp, yet each inefficient; the first were growing young lads, from 16 to 19, the second, men supposed not to exceed 35 years of age; many of them, however, were nearer 50, and some beyond that age. But even at 35 a man is too far advanced in life to make a soldier.

"The consequences were soon manifest. These lads, exposed as they were to the hardships and privations of life, rapidly succumbed. Many died at the seat of war; many more were sent home after a few weeks' or months' service—some to die, others to be invalided. A good many, however, whose constitutions were not seriously impaired, joined their depots, and if allowed to attain maturity before they are again exposed or overworked, may yet become effective soldiers. As for the other class, men who had lost the elasticity of youth, and whose habits were already formed, they found the education and discipline of a soldier too irksome, and many of them feigned or exaggerated ailments, for the purpose of getting discharged. It was useless attempting to retain such men—they never could be made soldiers.

"The contrast between the old and young armies of the Crimea was very marked. The great majority of the first arrivals were fine men, in the prime of life, though sadly reduced by disease and privations. But how different were the invalids who arrived at a later date; some of them old and worn out, though of only a few months' service; others half grown, sickly-looking men, who should never have been enlisted; but they were more to be pitied than condemned for having undertaken duties, the nature of which they were ignorant of, and physically unable to perform."

We know not what degree of importance will be attached to experience of this nature by our government and the chiefs of the military department; but it is full of instruction as a matter of military hygiene, and as such is applicable to the army of any country, even though that country could promise itself the blessing of prolonged peace. No doubt a question of this nature was in a good measure solved by the American troops in their march to, and subsequent encampment in, Utah.

A full account of the several diseases under which the British army suffered during the war in the East is given in sections four, five, six, seven, eight, and nine of the second volume of the "History." Section third contains a "General Sketch of the Course of Disease," and section tenth is on "Mortality" among the troops. It will be seen that in our preceding remarks and digested summaries, we have not followed the order of sequence laid down in the volumes before us, but have grouped subjects as seemed to us most convenient. In the too limited space which remains for us, we shall make our dottings in the line pursued by the reporter, Dr. Hanbury. We are told that nearly all the diseases by which the army was afflicted during the late war were of a kind more or less incidental to troops employed on active service in the field, and familiar to the conditions of camp life. From time immemorial fevers and fluxes have represented the diseases of armies and of campaigns, and their occurrence in the present instance was merely remarkable for the amazing prevalence and mortality which, for a considerable period, they obtained.

*Cholera.*—In reference to *cholera*, it is remarked that hitherto its appearance had been entirely an exceptional occurrence, the pestilence confining itself to small bodies of troops in camp or on the line of march, appearing only for short periods, and generally in detached positions, but seldom constituting itself an agent of wide-spread destruction. Now, however, we have to chronicle a disease which at an early date assailed the army, at first in Turkey, and afterwards in the Crimea, for a period of nearly two years, followed in its footsteps, and took up an almost permanent residence in the camp; at one time subsiding into comparative insignificance, and disappearing, to break forth again with redoubled fury and destruction. Looking at the history of cholera, and the late experience respecting this pestilence, there is reason to fear that it will assume a fixedness of endemic character in the different countries of Europe, similar to that which it holds in the various provinces of Asia, or more definitely of British India. A new element, the appearance and extent of which it will be difficult, if not impossible, to predict and measure, will thus be introduced in modifying the military results of a campaign. War will then become a game of much greater uncertainty than it has ever yet been, and victory will not necessarily incline to the side of the strong and the most skilled in strategy. Cholera presented itself on two occasions in the British army during the war, in an epidemic form; the first began in June, 1854, (in Turkey,) and for three months increased in prevalence, and then declined, subsiding slowly, and in an irregular manner, until the month of February, 1855, when it quite disappeared. The following month marks a clear interval between the two epidemics, for no case of the disease was recorded in the army in March, 1855.

In the history of the first of these two epidemics, details are furnished of the localities and spots in which the disease broke out, and of the order of succession in which it made its attacks on different bodies of troops. The disease was said to be imported into Bulgaria, and a plausible narrative of its introduction related by Colonel Neale, the British consul at Varna. The order of transmission is said to have occurred in the following manner: French troops from Avignon had the cholera among them when they embarked at Marseilles for Varna; some cases occurred on board during the voyage. At Varna, the disease "spread progressively through the town and allied forces, attacking the French and Turks simultaneously, and afterwards the English, no class of people, no description of locality, obtaining an exemption from it." On the other hand, we read, that when cholera was reported to have made its appearance in the French portion of the general hospital, which had been an old Turkish barrack, consisting of two quadrangles, additional means of segregation to those already practiced, were adopted, but in vain, for the protection of the English troops which occupied one of the quadrangles. The communication from the English quadrangle with the common passage had been boarded up, and a gate opened at the opposite or north side of the square. Notwithstanding all these precautions, a man was seized on the night of the 20th of July with the disease, on the ground floor, on the right-hand side of the entrance gate, the most distant point in the whole building from the French hospital, and he died in a few hours. The following day another man was attacked in the same ward, within three beds of the first case, and died in the afternoon. He had been eighteen days in the hospital, and was convalescent from fever. On the 9th of August, the disease in its most malignant form simultaneously appeared on board the "Britannia," "Albion," Trafalgar," "Tribune," and other vessels of war. Dr. Hall inclines to a belief in cholera having had its origin in local circumstances and peculiar atmospherical changes. Dr. Downes attributes the appearance of the disease among the French troops in the Piræus, (the harbor of Athens,) to importation by a French steamer. On this point it is worthy of remark, as attested by M. Baudens,* that, during the war, scarcely a week elapsed without persons with cholera having been brought to Constantinople by steam-vessels, and yet the disease did not prevail among the Mussulman population.

*Diarrhœa,* which preceded and accompanied cholera, was scarcely less exhausting and fatal than cholera itself. From the month of July, when the latter began, and during its entire epidemic course, diarrhœa prevailed; it affected the men of regiments in which cholera but slightly appeared, and there was scarcely a man who escaped an attack of this affection, while a vast proportion of the army was habitually affected by it; and no sooner were troops landed in the Crimea, than this complaint became almost universal among them. The effect was to reduce the physical stamina and vigor of the troops, and while lowering their standard of health, greatly, of course, to impair their efficiency. They were no longer able to undergo the same exertion, were fatigued by short marches; the men fell out of their ranks in great numbers; they could not in many instances carry their burdens, and their canteens and even blankets were thrown away.

The greatest sufferers from the choleraic poison were the regiments and troops newly arrived. This was observed in those which reached

* Souvenirs d'une Mission Médicale a l'Armée d'Orient.

Sebastopol in the early part of the winter of 1854–55, as well as in those which landed during the prevalence of the second epidemic in 1855. It would seem to have been established as a rule marked with few exceptions, that from the unacclimated, the recently-arrived drafts, cholera selected its chief victims.

*Consecutive Fever* was a frequent sequela of cholera, and in many cases was followed by death. The ratio of mortality from cholera was very great; and, different from what has been generally observed in civil practice, the proportion of deaths to the number treated was as large after the pestilence had considerably subsided as in the earlier period of its outbreak. Some explanation is offered in the fact that in no case did the medical officers make returns under the head of cholera, unless the symptoms were well pronounced and pathognomonic of the disease. There was an outbreak of cholera at Scutari (near Constantinople) in the latter part of the year 1855, sudden and abrupt in its invasion, but extremely short-lived.

*Etiology of Cholera.*—A number of facts are introduced, which seem to show the influence of locality in the production and prevalence of this disease. To a great extent the *sanitary* character of a particular place was decided by the degree to which cholera extended in it, "for in disease the effects being known, nothing is easier than to predicate the causes." But, as the reporter justly observes, "those individuals who most dogmatically assert the cause after the event, are unable to predicate the effects with any near approach to accuracy from a consideration of ostensible causes, nor are they competent to explain why these fall short of, or exceed their estimate, or why they do not express themselves in the precise manner, and for the exact time which may have been anticipated." A strong lesson of caution in drawing conclusions as to predisposing causes is given in the following curious and instructive fact:—It seems, "that although the localities in which cholera broke out among the troops in Bulgaria appeared, in a sanitary point of view, extremely objectionable, although the character of the climate, the nature of the soldier's accommodation, duties, diet, etc. seemed to favor the extension of the pestilence, yet it prevailed to a greater degree in the 4th Division of infantry, which did not land in Bulgaria at all, than in any other portion of the army; further, the disease thus assailed the 4th Division in a more severe form, not alone while the troops were on board ship, but as it marched side by side with the other divisions of the army from 'Old Fort' to the heights before Sebastopol." It was observed, however, in confirmation of that view which exhibits cholera as dependent on atmospherical conditions, that in the attack at Scutari, "as well as in every other instance which occurred, the outbreak of the disease seems to have been with changes from dry weather to a humid state of the atmosphere."

Little need be said of the treatment of cholera in the different corps assailed by the disease, for nothing encouraging, on this head, is added to our existing knowledge in the work before us. Of the deaths from cholera, it is said in the report that they amounted to 4513 during the war; and that the mortality from all other diseases (exclusive of wounds and mechanical injuries) was 11,785. The proportion, therefore, which the deaths from cholera bear to those of all other diseases, was 38·2 per cent., or more than one-third. Of this mortality there occurred 2903 deaths in the first epidemic, and 1610 in the second epidemic.

*Diseases of the Bowels* obtained a degree of prevalence for eighteen months, during the Crimean war, unknown in British military annals, "and for one-third of that period presented themselves in an aspect more fatal than has ever before been recorded. And while the causes to which the great extension of the disease may be considered as mainly represented—first, by an epidemic or choleraic constitution of the air and exposure of the unseasoned soldier to the endemic agencies of a hot climate, in the summer and autumnal months; and second, by the hardships, privations, and sufferings of a winter siege, carried on in an inhospitable climate, those of its mortality were chiefly confined to the latter—the hardships, etc. of the siege, at such a season." Bowel affections, at first in Bulgaria, and afterwards in the Crimea, prevailed almost entirely in connection with cholera; but in the last two months of 1854, and the first of 1855, "the circumstances of the soldier's life, the nature of the service, and the severity of the climate, were pre-eminently calculated to induce this class of diseases, and the effects were disastrous beyond all example, and resemble more the accident of fate than the adopted result of self-imposed position." It may be asserted that the army, at least the infantry force, represented a stricken mass. There was, indeed, scarcely a sound man in the ranks, with the exception of a small number recently arrived in the country. The number admitted represented the capacity of the hospitals rather than the necessities of the time, for, in fact, the men in the trenches, and daily proceeding on fatigue duty to Balaklava, were, in a great proportion, suitable candidates for admission into the hospitals. A lesson of etiology, and of treatment of these diseases, and also of patriotism, is furnished in the following sentence. The reporter had just been speaking of the sympathy of the British public having been for a time frustrated, in its anxiety to discover some specific cause, some panacea for the wide-spread suffering in the ranks of the British soldiers. Fully convinced were the medical officers, "that there was nothing recondite or mysterious in the causes of the frightful losses which were being daily incurred; and the result more than justified their conclusions; for when at length the gushing sympathies of the people found a proper channel for their expression, and the supplies of clothing, bedding, and food, so largely provided by the government, reached the army, the soldier forthwith, under the fostering care of the nation, (of which there is not on record a greater or more noble instance,) advanced to a state of high efficiency, and in a few months reached a standard of health which it was the more delightful to contemplate, as it was so unusual, and, *in general*, so totally unexpected."

In the month of February, the diminution of disease corresponded with the amelioration in the severity of the season, abundance of personal clothing, and better accommodation in tents and bedding, food of somewhat better quality, and regularly cooked. The number of admissions, which in January was 5436, was, in this month, reduced to 2336; and in the month of March it became evident that these deadly fluxes had nearly ended. The total number of cases of bowel diseases treated in hospitals during the war was 55,765, or 34·2 per cent. of the whole number of cases admitted from disease during this time.

*Diarrhœa.*—To diarrhœa, associated with cholera, is to be referred one-fourth of all the casualties which occurred from wounds and sickness during the war. The deteriorating and destructive influence of this combination on the health and military efficiency of the troops has been described in a preceding page. Diarrhœa, dependent on endemic causes—great heat by day, coolness and heavy dews at night, lying on or near the damp ground, the use of unwholesome spirits, improper food, including unripe fruits and green succulent vegetables—spread very little in Bulgaria or the Crimea, and was, therefore, comparatively unimportant. Diarrhœa, the immediate result of hardships, exposure, and defective diet, could not be well studied, apart from its common association or blending with the choleraic variety; nor was it easy to say how far these causes would have been operative in common circumstances without the predisposing state of enfeebled constitution and scorbutic taint. We quote the following as suggestive of a pathology and treatment which a practitioner may find applicable to cases out of camp:—

"It was not unusual to see men affected with this '*congestive*' form of diarrhœa, who had only been a short time with the army, and it was the most severe description of the affection, and the most rapid and urgent in its symptoms, which occurred. It seemed proximately induced by the interruption of the cutaneous and biliary functions, and congestion of the abdominal viscera and portal veins; and while it was almost instantly relieved by rest and warmth, it was extremely apt (the exciting causes being continued) to merge into dysentery, and the dysentery thus originating was prone to pass on to more rapid disorganization than when otherwise induced."

Next in importance to the inculcation of a few physiological truths, as a foundation for hygienic practices, ought to be that of the importance of rest and warmth in the forming stage or inception of all the forms of congestive disease, including not only those now under consideration, but also cholera, fevers, and typhoid pneumonia. A timely yielding to the threatened storm will not only prevent much future racking, but also entire uprooting and destruction.

*Scorbutic Diarrhœa.*—In the report before us, diarrhœa is examined in its relation to the operation of causes still more complicated, and the state of the system which these causes determined. Concurrently with the immediately injurious effects of agencies already enumerated, there were caused by these latter a reduction of the system, an impairing of the functions, a vitiation of the blood, and a lowering of vitality. "Diarrhœa occurring in the pathological state of the system thus induced, presented important modifications, which were determined rather by such state than by any of the individual causes now mentioned, or any combination of them, and it was of course chiefly remarked in men who experienced great hardships, privations, and sufferings for a considerable period. In this form of the affection, the patient presented himself in a feeble, emaciated state, having previously suffered from occasional attacks of diarrhœa, and perhaps from other complaints. There was loss of appetite, loathing of food, apathy of mind, debility or disinclination for exertion; the pulse was small, slow, and feeble; the skin was dry and rough, often of a dusky, muddy-brown color; that of the abdomen was retracted, covered with dry, hard scales; the features assumed a wan and listless expression; the face and eyelids were œdematous; the mucous membrane of the mouth and lips became exsanguined, the legs and feet were generally swollen, and there was felt in them a sense of numbness and coldness. The tongue appeared large and flabby, or was covered with a slimy mucus, and it was often indented by the teeth. The dejections were voided frequently, and sometimes with a degree of straining and pain; they were occasionally serous and watery. Sometimes they were of a clay color, or grayish, and extremely fetid, but these last characters usually occurred in the worst cases, and those attended with organic lesions. The appearance of bile in the dejections was not constant, and it seemed rather to be periodically thrown off as an excretion, than to be expurged as an *element* which had been applied in the digestive process; it was seldom observed of a depraved quality in the evacuations, except in the complication of a low adynamic fever, which was by no means an infrequent occurrence. Notwithstanding the grave nature of the symptoms here detailed, diarrhœa was yet, under careful treatment, and with suitable resources, still a very unmanageable disease." The scorbutic nature of the affection was evinced by livid and swollen gums, and their bleeding on slight pressure; petechial spots and ulcers on the legs; patches of ecchymosis on the calves of the legs; listlessness; dyspnœa, on slight exertion; a feeling of aching pains in the limbs, with a sense of coldness; œdema of the feet, and sudden hemorrhage from the bowels; vomiting of blood, discharge of blood from the fauces, epistaxis, etc.; and occasionally the scorbutic nature of the disease was still further shown by fatal sinking, and effusions into the pericardium or pleuræ. The low vitality with which diarrhœa under these circumstances was associated was often illustrated by the occurrence of frost-bite. Of the treatment of diarrhœa nothing new or positive is said in the report, nor is confidence expressed in the result of any mode of medication adopted.

*Dysentery* is declared to be "*primarily*" a disease of tropical climates and of cold latitudes; and it is added, that its prevalence in both is determined, in a great measure, by the evidence of the locality and nature of the season, and that the *conditions of life* which favor its development and extension in the soldier, are represented in the nature of the clothing and bedding, the amount of exposure and fatigue, and the character of the food with which he is provided. This disease is undoubtedly, also, observed as an effect of special causes, bad water or other agencies, the nature of which it is often not easy to appreciate; and troops have suffered from the complaint when its appearance could scarcely be assigned to anything more definite and tangible than the state of the atmosphere. That dysentery should easily be developed under the circumstances of a soldier's life is easily understood.

Dysentery attracted but slight notice while the army was in Bulgaria, (in the summer of 1854,) and made little progress before Sebastopol, until the month of November, between which time and the end of January it continued to be extremely prevalent and fatal. We are told that "the prevalence and mortality of dysentery, from the date the army landed on the Crimea until the end of February or March, from month to month, almost accurately represents the increase, culmination, and decline of the wants of the service—of the sufferings of the soldier; with these they move in a parallel course, for in proportion as they were present so did they constitute the cause of the disease, and the influence of the climate in increasing or diminishing the effect was probably nearly commensurate with its influence in increasing and mitigating the hardships and sufferings of the troops." The progress of the disease in the army from the month of May, 1855, was perceptibly advanced under the influence of change of season. The highest number of admissions into the hospitals, viz., 817, was reached in July; in August and September, the number of cases treated amounted to 621 and 564; but from the end of this latter month dysentery ceased to be a disease of importance. The progress of the complaint was much less certain and definite than when it arises from causes less numerous and complicated. We have not room for a detail of the symptoms of camp dysentery, which was generally preceded by diarrhœa. The most certain signs of danger were a peculiar aspect of the countenance, which it is difficult to describe, but is yet known to attend upon certain stages of intestinal lesions, pungent local heat of the abdomen, generally of the hypogastric region, and fetid and unnatural character of the evacuations. In the early stage of asthenic dysentery, that which almost invariably prevailed in the Crimea, there was very little change of the pulse, or symptomatic fever; but when the disease extended beyond the inner or mucous membrane, and disorganization had begun, the pulse became small, quick, and concentrated. If the skin were dry and hot, and partial sweats appeared occasionally on the head and chest, the extremities or feet became cold, the sufferer was in imminent danger. Dysentery was frequently associated with fever of a low or putrid type, which interrupted the course of the disease and altered its symptomatology. The presence of scorbutic complication with dysentery was indicated by the symptoms proper to scurvy. The state thus induced was one of peculiar obstinacy, and rendered its subjects very liable to relapse. The greater tendency to frost-bite in dysentery was as well marked as it was in diarrhœa. We shall reserve what may be said of the *post-mortem* appearances in these bowel affections for another time, when we can analyze the "Report on the Pathology of the Diseases of the Army in the East." The treatment of dysentery may be dismissed in a few words. It was, "of necessity, extremely defective." The first step was to restore, as much as possible, the patient to the ordinary conditions of life from which he had been removed. With this view, the requirements of rest, warmth, cleanliness, free ventilation, sufficient cubic space, and a varied nutritious diet, formed the chief indications to be attended to. The most valuable remedy, in general estimation, was opium; but it had to be used with caution, "for if pushed too far it lowered the circulation and predisposed to gangrene in the parts most distant from the heart." Lime-juice was found to be very serviceable in those cases in which dejections of indigested food were observed, and also where the bowel affection was associated with scorbutic taint of the system. The dose was from one to three ounces, with sugar and water, two or three times a day.

The mortality from diseases of the bowels in the British army was mainly dependent on the particular circumstances of exposure, want, and fatigue. Climate, *per se*, had little to do with it. Thus it is related that of the whole number of deaths returned, viz., 5950—5301, or 89·0 per cent., occurred from the period which elapsed between the opening of the siege of Sebastopol, on October first, 1854, and the termination of the following April; while in the months of December, January, and February, taken together, 4145 deaths occurred; and in January alone, 2033, the latter, in itself, constituting a mortality considerably greater than was incurred by wounds during the war.

*Fever.*—Our waning space reminds us of the necessity of passing, without delay, to a consideration of fever under the interesting aspects in which it presented itself in the British army, at first in Bulgaria, and afterwards in the Crimea, and which are described in the "History." Climate, season, and localities, imperfect protection from atmospherical extremes and vicissitudes in bell tents, without a bedstead, and entire want of ventilation, added to harassing field-day duties, were all causes which are recognized as equal to the production of fever among the troops who were thus exposed. The final result was not only considerable mortality in Bulgaria, but ineffectiveness of a large portion of the army. When, late in the season, the troops left Turkey and arrived in the Crimea, the fever rapidly subsided until the end of November—a change not to be explained by the nature of the duties of camp life, which, at first in marches to Sebastopol, and then in the fatigued exposure during the first period of the siege, were more arduous and exhausting than ever. The relief was due mainly to the beneficial influence of the removal of the troops from an ungenial climate and insalubrious position during an unhealthy season of the year. Remittent and continued fevers were the most prominent of those which appeared in Bulgaria, both on account of the numbers presented and the exceedingly severe nature of many of the cases, and the formidable symptoms which arose during their progress. These fevers were all of the asthenic form, and presented almost invariably a disposition to local complications. There were determinations either to the head or to the abdominal viscera, and occasionally to both. Deranged secretion of the liver was very common, which showed itself in hyper-secretion of bile; gastro-enteric symptoms were still more usual, even when the discharges from the bowels did not indicate deranged hepatic function. The remissions in fevers of the remittent type were often very imperfect and irregular, and sometimes it was lost and merged in the continued form. Seldom were the "solid viscera" seriously compromised, whether the fever assumed the intermittent, the remittent, or the adynamic form. In scarcely an instance was the spleen affected, and the change in the liver seldom went beyond congestion. There seemed to be no definite period of duration of the disease. Emphatic testimony is borne to the efficiency of prophylaxis, by a removal of three Divisions of the army from the filthy neighborhood of Varna and the marshy grounds near the Devna Lake to the plateau on which Monastir stands, and to the heights near the village of Govrekoi, and the elevated ground in the Adrianople road south of Galata Point.

After presenting the principal features which characterized fever in the army of the Crimea, from September, 1854, to the end of the following April, the reporter makes the following remarks: "At first of an unimportant character, induced by exposure to atmospheric changes and other ordinary agencies, it generally assumed a mild form, marked by bilious and gastric derangement, and complicated with diarrhœa. At a later period, in December and January, the great hardships of the service, cold and wet, constant night-watching, insufficient clothing and bedding, and food of defective composition, rendered dysentery a prevalent and destructive disease, and we find that fever was often superinduced upon it, and frequently succeeded by it; and lastly, fever occurred as a consequence of a period of hardship, exposure, and privations, became epidemic, acquired contagious properties, presented the form of remittent typhus of camps, and periods of famine and distress, and was distinguished by moderate disposition to relapses." The fever was not accompanied by disorganizations of the solid parenchymatous viscera, as the liver or the spleen, and dropsy was not often observed. Convalescence in the last four months of the existence of the fever was extremely protracted and unsatisfactory, and the tendency to relapse great. Relapses were referred to three causes: 1. A vitiated state of the blood, and a low, depraved, cachectic condition, induced by long-continued hardships and privations. 2. A residence in the vitiated air of hospitals, the tainted atmosphere of overcrowded tents, which introduced morbific elements into the circulation. 3. Organic changes of a complicated kind in the small and large intestines. It is most probable, however, that these lesions themselves were but an evidence of a morbid state of the system. The really contagious character of the fever is not so clear, if we are to adopt the view entertained on this subject by Dr. Crawford, who, in his report, while asserting that the fever is unquestionably contagious, adds: "But a residence in the same atmosphere with patients suffering from it seems necessary for the production of the disease in a healthy individual in this way." This would show the fever to be infectious, the product of a contaminated air, as there is every reason to believe that yellow fever is, but not communicable from person to person, as is the case with a fever truly contagious. The distinction is an important one, and its disregard has led to much confusion and misapprehension, to needless panic, and cruel neglect of those attacked with fever. If we ventilate thoroughly the wards of a fever hospital, we may smile at the idea of contagion in its wards or of farther propagation of the disease from them. Alter the conditions of the soil in yellow-fever regions, and this dreaded pestilence will disappear for want of those emanations which, in conjunction with a high temperature, give rise to it.

In the Report before us we read: "The disposition of fever to extend itself was not observed among troops whose physical stamina had not been much reduced—as the cavalry and the artillery—and was much greater in large than in small hospitals, and was especially manifest in some localities; but when it was observed in a regiment, recruits were liable to contract fever."

Among the prophylactic, and at the same time curative measures recommended by the medical staff, one was found to be quite efficacious. It consisted in the provision of a certain number of steamers suitable for the reception of the sick, and transferring the latter in a regular and systematic manner to the various hospitals of the Bosphorus. A second measure was the opening of the general hospital near the old Genoese Castle, overlooking the entrance into the harbor of Balaklava. We find but little said of the morbid lesions to illustrate the pathology of the fever of the Crimean war; but we shall reserve at any rate what is procurable under this head for another occasion, when greater justice can be done to the subject.

The treatment of this fever consisted mainly, in the first instance, in ventilation and cleanliness, by which means, if due attention were early paid, the disease was prevented from running into a low, putrid, and adynamic type. Rest and warmth were also necessary here, as they were found to be in bowel affections. No curative measures were of greater importance than the selection of suiable diet. "In the more severe cases, arrow-root, milk, essence of beef much diluted, and weak lemonade were most generally prescribed, and wine was often administered to the extent of six or eight ounces, while brandy was occasionally found necessary in considerable quantity; but in cases less grave, the diet was still more liberal, and comprised the use of animal and vegetable broths, and during convalescence beer was found extremely useful. Milk, as a remedy to check vomiting, which was very frequently an extremely obstinate symptom, proved more effectual than any other article of food and than any of the numerous medicines which were administered for this purpose." But, in order to derive full benefit from a suitable diet in the treatment of fever, it was necessary that the articles specified should be taken in the smallest quantities. The treatment of fever here laid down is not that of the schools, nor is it one which will be called for in ordinary private practice; but if it suggest an imitation even in single cases where constitution, exposure, and defective food have assimilated our patient to the soldier, it cannot fail to do good. In hospital practice in civil life, we not unfrequently find wretched creatures, exhausted by age, intemperance, and defective nutrition, who require to be treated in a manner very analogous to that which was ascertained to be most successful in the troops before Sebastopol. Medicines, of whatever kind, and in whatever mode of combination, it is justly remarked in the report, were entirely subordinate to the means above indicated. "A mild emetic was sometimes prescribed in the early stages, and an alterative of gray powder and ipecacuanha given and repeated with advantage; but in the progress of the symptoms, stimulants, diaphoretics, and the whole class of cardiac remedies were considered most necessary. Among these may be mentioned a mixture of aromatic spirit of ammonia and sweet spirit of nitre with camphor. The constant occurrence of vomiting, or hiccough, demanded the use of hydrocyanic acid, chloroform, morphia, and effervescent draughts; further, diarrhœa being an extremely obstinate complication, occurring at intervals, required to be checked by the use of opium and astringents." Considerable management was, however, necessary in the extent to which these remedies were used, for a sudden check of the diarrhœa was often followed by aggravation of the fever. Congestion of the lungs, and low typhoid pneumonia, which were so constantly observed, were combated by the application to the chest of blisters, sinapisms, and stimulating liniments; while blisters and irritant ointments were often applied to the neck and parts of the scalp, when the brain became involved in the course of the disease. Great caution was necessary in the use of these external applications, owing to the low state of vitality and the depraved state of the blood, so that blistered surfaces sometimes healed slowly, and sloughing, which it was difficult to arrest, occasionally occurred.

The mortality from fever during the war was 3446, and the admissions into the hospitals for the same period amounted to 31,204, the former being to the latter in the ratio of 11 per cent., and representing 19 per cent. of the mortality from all causes during the war.

*Scurvy*, associated in the minds of both the professional and general reader with sea life, and restriction for a long period to salt provisions, figures somewhat largely in the medical histories of military life, where the troops have been confined within narrow limits, either as besiegers or besieged, exposed to cold and moisture, and deprived of a due supply of fresh wholesome food and vegetables. In the section on this disease we read the following, which in a short compass conveys a clear idea of the extent to which the scorbutic diathesis prevailed in the British army, and the coloring which it gave to the current diseases:—

"From the details now submitted, it will be understood that scurvy was an affection of some importance, at one period, in the army. It is to be understood, however, that the returns convey but a very faint conception of the disastrous part which it acted among the troops; for, although it was only in comparatively rare instances that it presented itself in well-defined forms, and as an independent affection, yet the prevalence of scorbutic taint was wide-spread, and in a vast proportion of cases evident indications of it existed as a complication of other diseases—fever and affections of the bowels. Indeed, it may be stated that during the first six months of the siege, all morbid ailings in the older residents were more or less distinctly marked by scorbutic symptoms; and the fact is constantly commented on by medical officers. Thus Dr. Marlow, 28th Regiment, remarks: 'Although there are apparently few cases of pure scurvy marked in the return, nearly every admission into the hospital exhibited unequivocal signs of the scorbutic taint.'" Many of the characteristic features of scurvy have been already enumerated in speaking of the complication of this disease with bowel complaints. One of the most constant precursory symptoms of scurvy was an obscure form of muscular rheumatism; the individual complained of pains in his legs of an aching character, and his movements were tedious and painful; but in no case was there articular inflammation. This disease, as would appear from M. Baudens,* occurred to a greater extent and assumed still more serious characters in the French army, during the autumn and winter of 1855–56, than it did in the English army during the preceding year; and the facts prove irresistibly that certain conditions of life, defective nutritious diet, imperfect shelter and accommodation, over-crowding, and filth, as distinct from excessive labor and fatigue, were the essential causes of this disease, and of the fatal epidemic of fever which succeeded it.

The measures of a prophylactic nature recommended in an urgent manner by the medical officers of the army were little heeded, or we ought rather to say, could not be carried out by those in command, owing to the negligence and blundering in the commissariat department. For a long time after a supply of vegetables had arrived at Balaklava from England, the utter want of means of transport prevented them from reaching the troops, who were in sight of what was destined for them. "The treatment was to a great extent expressed in the use of vegetables, lime-juice, milk, fresh and preserved meats, soups, bread, wine, and beer; the use, in short, of a varied and nutritious diet." It is important to notice that preserved

---

* Op. cit.

meats, soups, and vegetables were deemed nearly as valuable as these articles in their fresh state. The preparation of preserved or pressed vegetables which gave the greatest satisfaction, were those made of "mixed" vegetables, prepared according to the French method, and known as "Morel's." It may be added that preserved potatoes, whether in the shape of dry crumbs, or shreds like maccaroni, were not only much relished by the sick, but were considered extremely valuable in scorbutic affections. Some difference of opinion occurred regarding the value of lime-juice as a remedy in scurvy. We cannot, however, fail to appreciate its usefulness in assimilating salt meat and biscuit, the common food of the soldier at this time, and thus removing, physiologically, a potent cause of fluxes. There follows, under the head of "Concluding Remarks," some instructive suggestions on the victualing of troops in the field.

The section given to *Frcst-bite and Gangrene* conveys very valuable information, by presenting what at first would seem to be a merely incidental casualty as a formidable variety of disease, affecting a large number of men. The total number of cases noted in the returns is 2390, and of these, 1924 occurred in the first winter, 1854–55, and 474 in the second. The total number of deaths was 463, and all occurred, with the exception of six, in the first winter of the siege. "It was not noticed that a low range of temperature exclusively determined the existence of this affection. On the contrary, it was observed that many cases occurred as a direct consequence of exposure to wet, and more especially when it was accompanied by winds from the northeast. Further, frost-bite very frequently occurred during periods of thaw; but, above all, was it a common event of a thaw by day alternated with frost at night, for on this state of the weather the clothes of the soldier were at once saturated with wet, and again stiffened into ice." The circumstances which rendered the action of cold so unusually severe, were the duties which devolved upon the men, the inadequate nature of their clothing, and the miserable accommodations in tents and bedding. Bearing these things in mind, we can better appreciate the view which has been taken on this subject by Dr. Dumbreck, who says: "I consider that many of the cases denominated *gelatio*, were in reality gangrene from debility; many of the men having been attacked when the thermometer was between 40° and 50° Fahrenheit." We shall probably take the most correct view in regarding the disease as the compound effect of cold and a reduced state of the system—the gangrene of cold and debility.

*Diseases of the Organs of Respiration*, as they were met in the British army, form the subject of section ninth of the medical history of the war. Contrary to what might have been anticipated, this class of diseases was met with to only a trifling extent. The "whole number of cases treated during the war amounted to 12,382, and of deaths to 644, representing 7·6 per cent. of the total admissions, and 3·9 per cent. of the mortality from all diseases; and when it is considered that under this class of affections are included nine diseases of very common occurrence in the ordinary circumstances of life, it will be at once understood how comparatively insignificant was the part it acted among the troops." The total number of cases assigned to *pneumonia* was 590, of which number 161 proved fatal; 159 of these cases were admitted in the first winter and spring, between November, 1854, and April, 1855, of which 79 were fatal; and 257 were admitted in the following winter and spring, of which 45 were attended with fatal issue; the remainder of the admissions, 208, and of the deaths, 39, were distributed over the summer and autumn months of 1854 and 1855, 39 cases, however, having been admitted in May and June, 1856, of which five terminated fatally. There was a very noticeable difference in the proportionate mortality in the two winters, that in the first being 63·1 per cent., and in the second 16·7 per cent. of the number admitted. The form which this disease assumed in the army was almost invariably of the low asthenic kind, with extreme tendency to pass rapidly into typhoid states; and blood-letting or other depletion was, therefore, seldom resorted to in the treatment. A common association of pneumonia was with affections of the bowels and fever in very reduced states of the system, and hence a lack of any decided or well-marked indications of the true character of the disease, so that a slight cough and hurried respiration were sometimes the only discoverable circumstances. Sometimes pneumonia was secondary, occurring in the worst stages of bowel complaints and of fevers, in which it was the immediate cause of the fatal issue. The *post-mortem* lesions will engage our attention at another time, when the subject of the pathology of the diseases of the army in the East comes up formally for notice and consideration.

*Phthisis Pulmonalis* was of rare occurrence, and the whole number of cases, during a period of twenty-six months, did not exceed 185, notwithstanding the large number of boys in the army, and the known predominance of diseases of the pulmonary organs among Englishmen in early life. Many medical officers believed that the habit of living in the open air, under canvas, brought with it a tolerance in the soldier of those atmospheric changes which are so productive of derangement and disease of the pulmonary organs. "In truth, it may be asserted that diseases of the lungs are rather the *heritage* of civilized life, and of *artificial* training, while the fluxes are the more appropriate effects of those *nomadic* and *primitive* states of existence, the requirements of which are fulfilled in the shelter of the tent, or of rude arbors constructed of reeds or the branches of trees."

*Catarrh, Acute or Chronic*, furnished 10,083 cases, of which 240 proved fatal. Of the entire number, 9506 were referred to the acute, and 577 to the chronic form. The mortality in the months of December, 1854, and of January, February, and March, 1855, was 183, or 76·2 per cent., while only 12 fatal cases were recorded in the same months of the following year.

*Bronchitis* presented itself with less frequency than catarrh, but more so than the other affections of the lungs; the total number of cases received under treatment having been 1111. Although it acquired nearly an equal degree of extension, regard being had to the strength of the army in the early part of the siege, and in the winter and spring of the following year, its mortality was almost exclusively exhibited in the winter and spring of 1854–55. Bronchitis proved to be not only a very fatal disease at this season, in the few instances which were returned, but very frequently it occurred as a grave complication in the more serious cases of fever, and was the immediate cause of the fatal issue. The symptoms were insidious, both in their commencement and progress, and manifested great want of power. Accordingly, constant watching and frequent examination, with a view to detect the earliest evidences of the graver symptoms of the affection, and the employment of blisters and other suitable remedies, from the moment serious internal mischief was threatened, were essential conditions for its successful treatment.

Dr. Hanbury, Staff Surgeon, author of the History of Diseases as they occurred in the British army during the war, has acquitted himself of his task in a most satisfactory manner. It has been our sole aim to place before our readers a condensed sketch of the details and descriptions introduced by him, as well as of the conclusions at which he arrived, after a wide and patient survey of the whole field of operations. Seldom will there be found so large a body of medical history, and especially of etiology and clinical medicine, and of preventive hygiene, with so little speculation and hypothesis. This excellent feature of the work has rendered, however, the labor of analysis and condensation more difficult than under the ordinary circumstances of composition. The amount of tabular matter is great, and would of itself constitute a work of valuable reference.

Respecting the successful issue of the trade and game of war, it is justly remarked by Dr. Hanbury, in the closing paragraph of the "History," that, as carried on according to the enlightened notions of humanity which distinguishes modern civilization, war has, in truth, become in a great degree a problem of sanitary science; and the greatest genius for command in battle may issue in less successful enterprise than the sagacious and provident arrangement which would provide for the continued preservation of the soldier in health and efficiency. It follows that every officer, taking a more extended view than heretofore of his duties, must feel the importance of a general knowledge of the causes affecting the health of his men, and of the measures most effective for preserving them in a proper state of physical vigor.

